# A whirlpool of emotion: How entrepreneurs’ empathy affects employees‘ emotional exhaustion

**DOI:** 10.3389/fpsyg.2022.933628

**Published:** 2022-08-08

**Authors:** Jiang Xu, Baobao Dong, Yinong Wang, Na Jiang, Yue Gao

**Affiliations:** ^1^School of Economics and Management, Changchun University of Technology, Changchun, China; ^2^School of Business and Management, Jilin University, Changchun, China; ^3^Changchun Humanities and Sciences College, Changchun, China

**Keywords:** entrepreneurs’ empathy, emotional exhaustion, psychological empowerment, emotion, new venture

## Abstract

Drawing upon upper echelons and self-determination theories, we hypothesize and test a mediating process linking entrepreneurs’ empathy to employees‘ emotional exhaustion and the moderating role of psychological empowerment. Based on a dyadic-survey study of entrepreneurs and their employees in high-tech new ventures in China, we conducted the empirical test by using hierarchical linear modeling (HLM) and found that entrepreneurs’ empathy has a negative effect on employees‘ emotional exhaustion, and psychological empowerment not only partially mediates the relationship between entrepreneurs’ empathy and employees‘ emotional exhaustion but also positively moderates the above relationship. This study frames an integrative perspective of emotions and psychologies and sheds a nuanced understanding of the mechanisms linking empathy with emotional exhaustion. Limitations and future directions are addressed.

## Introduction

With the breakout and continuous development of the COVID-19 pandemic, entrepreneurs and their new ventures are facing more challenges and threats and are struggling to survive (Tang and Gray, 2021). Besides, more and more employees in new ventures are experiencing emotional pressure or even breakdown at home and workplace. Because of liabilities of newness and smallness (Dong et al., 2020; Zheng et al., 2020), new ventures’ survival is largely dependent on the wisdom of their employees, a kind of valuable, rare, inimitable, and non-substitute resource causing competitive advantage for firms (Barney, 2001), supporting the competition and survival for new ventures. But one of the most dangerous episodes for new ventures is employees’ emotional exhaustion (Cropanzano et al., 2003), a peculiar psychological factor that means employees are endowed with excessive emotional demand but are unable to bear it when they interact with colleagues at the workplace (Wright and Cropanzano, 1998). Root causes of employees’ emotional exhaustion are often deeply embedded within the atmosphere of organizations (Pare and Tremblay, 2007). In light of these challenges, a stream of research has studied the factors that influence employees‘ emotional exhaustion (Walumbwa et al., 2011), among which the role of entrepreneurs is highly stressed (Dust et al., 2018). As shown in previous research, individual and group cognition, emotion, and psychology play a crucial role in the conceptual foundation of upper echelons theory (UET) (Carpenter et al., 2004; Hambrick, 2007). Far and wide, there is a deep understanding that cognitive and psychological models influence how individuals attend to, filter, and process information in a given situation. UET explains that these processes influence executives’ actions on followers and strategic themes (Hambrick and Lovelace, 2018). UET argues that leaders/entrepreneurs or top management teams are responsible for the development, and their characteristics and strategic choice behaviors can affect the daily operation, employees‘ emotions, crisis management, and performance of the firms (Hambrick and Mason, 1984; Hambrick, 2007; Scott et al., 2010). Importantly, recent calls in the emotion and psychology literature (Tata et al., 2017; De Cock et al., 2020) highlight that emotion-focused or trait-focused upper echelons perspective is becoming a more and more vital lens to explain the cause of employees’ emotional exhaustion.

Among the idiosyncrasies, entrepreneurs’ empathy is claimed to matter in regulating and balancing employees’ emotional exhaustion (Cooley et al., 2017; Tang and Gray, 2021). Empathy is defined as the human ability to put oneself in the place of another to better understand what that other person feels or thinks (Levy, 1997; Eklund, 2006). When facing distress, failure, anxiety, and frustration triggered by external environment or internal workplace factors, employees in new ventures will exert withdrawal behavior because of negative emotional perception (Allemand et al., 2015), thus causing depersonalization and reduced personal accomplishment and even more severe symptoms in their work (Ladebo, 2009). Only if employees get empathic concern from entrepreneurs, they may feel the leaders are sharing their feelings with them and showing perspective-taking for their psychology and mentality to improve their welfare (Vachon and Lynam, 2016). More precisely, unpacking the influential processes, we find that understanding employees’ needs and experiences and, thus, recognizing their emotions require leaders’ empathy, which shows that leaders should play a key role in harnessing employees’ emotions. Thus, vicarious empathy is a key factor in solving employees‘ emotional anxiety and exhaustion—both in facilitating employee passion and excitement in the workplace and enhancing their “recognition of an entrepreneurial problem-to-be-solved and in devising a more innovative and valuable solution” (Packard and Burnham, 2021, p. 9) in their daily work.

However, extant studies are limited in several ways. First, some research has already investigated the outcomes of empathy, such as employees’ organizational identification (Calvert et al., 2006), employee creativity (Sun et al., 2012), employees’ job evolvement (Kim and Richardson, 2003), organizational citizenship behavior (Stock and Bednarek, 2014), prosocial behaviors (Decety et al., 2016), and social entrepreneurial behaviors and activities (Toledano, 2020; Chatterjee et al., 2021). Some scholars have recalled the urgent need for the research about employees’ emotional exhaustion from the view of empathy of leaders to unveil its emotive micro-foundations (Bloom, 2017).

Second, the path of how leaders‘ empathy influences employees‘ emotional exhaustion is unclear (Vachon and Lynam, 2016). Some researchers argued that empathy, as a kind of emotive and psychological factor, must empower employees with a strong feeling of identification and encouragement to facilitate their positive behavior and capabilities, thus reducing their distress and then relieving emotional exhaustion (Wright and Cropanzano, 1998; Zhang and Bartol, 2010), as suggested by self-determination theory (Gagne and Deci, 2005). Therefore, by including cognitions regarding the meaning, personal competence, self-determination, and impact of work (Spreitzer, 1995), psychological empowerment is a motivational construct with integrative and active orientation and reflects intrinsic task motivation, which is a vital inhibitor of employees’ emotional exhaustion (Seibert et al., 2011; Hill et al., 2014). Accordingly, in this study, based on the self-determination theory, we examine the role of empathy of entrepreneurs‘ in promoting psychological empowerment in employees in their workplace, which, in turn, affect employees‘ emotions (e.g., emotional exhaustion, emotional high, or job stress reduction). That is, psychological empowerment as an integrative motivational mechanism can build a linkage between entrepreneurs‘ empathy and employees’ emotional exhaustion.

Third, despite past research efforts linking empathy to emotional exhaustion, a critical question remains unanswered, namely, to what extent does empathy affects emotional exhaustion? Empathy can have positive (e.g., via prosocial behavior, Fiori and Ortony, 2016) and negative (e.g., putting individual interests ahead of organizational interests may trigger cognitive overload, exhaustion, and bias, Antonakis et al., 2009) influence on emotions, respectively. The employees‘ capabilities of performing their work activities and controlling immediate work behaviors may affect the functions of leaders‘ empathy, that is, to improve or decrease their cognitive functioning efforts (Brown et al., 2005). How to regulate the effect of empathy needs more explanation in the view of psychological empowerment. As such, another task in the current study is to determine whether employee psychological empowerment functions as a boundary condition that strengthens the negative effect of entrepreneurs‘ empathy on employees’ emotional exhaustion.

**FIGURE 1:**
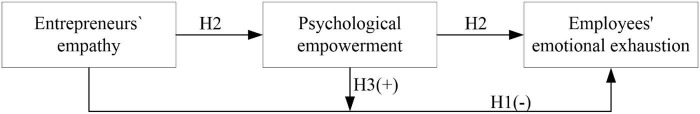
The conceptual model.

To address these research gaps, we draw upon upper echelons and self-determination theories to hypothesize that (1) entrepreneurs‘ empathy has a negative effect on employees’ emotional exhaustion, (2) psychological empowerment mediates the effect of entrepreneurs‘ empathy on employees’ emotional exhaustion, and (3) psychological empowerment moderates the effect of entrepreneurs‘ empathy on employees’ emotional exhaustion. This study contributes to empathy and emotional exhaustion literature and research in three important aspects. Most prominently, we supplement research on emotional exhaustion by introducing emotion-focused upper echelons and self-determination perspectives as nuanced angles to better understand employees’ emotional exhaustion in the severe contexts of new ventures. Besides, by clarifying psychological empowerment as a mediator in the entrepreneurs‘ empathy–employees’ emotional exhaustion relationship, this study extends previous research that has primarily examined the direct or main effect of empathy on employees’ emotional exhaustion (e.g., Bloom, 2017). Understanding the mediators of the above relationship sheds light on the mechanism through which entrepreneurs‘ empathy affects employees’ emotional exhaustion. Although the relationship between empathy and employees’ emotional exhaustion has been examined qualitatively, the present study is among the first to examine the effect of entrepreneurs‘ empathy on employees’ emotional exhaustion quantitatively in the context of new ventures. Finally, this research demonstrates that psychological empowerment is an integrative and active orientation that reinforces the motivational effects of empathy and may prove to be an important boundary condition on the influence of entrepreneurs‘ empathy (Figure 1).

## Theory and hypotheses

### Employees’ emotional exhaustion: An emotional challenge for entrepreneurs

There are several research investigating factors that determine whether employees’ emotional exhaustion is likely to be invoked (for a review, see Seibert et al., 2011). The emotional literature underlines that factors causing employees’ emotional exhaustion in new ventures remain poorly understood and that there is still a significant limitation in our understanding of employees’ emotional exhaustion and burnout (Halbesleben and Bowler, 2007; De Cock et al., 2020). We do not emphasize previous views which argue that employees’ emotional exhaustion is triggered by external factors (e.g., fierce market competition) that are out of control from employees (e.g., Phillips and Reddie, 2007), or that employees’ emotional exhaustion depends mainly on the imbalance of venture resource orchestration or mobilization (e.g., conservation of resources theory; Hobfoll and Shirom, 2001), or even the leaders’ ability to manage the teams and firms (e.g., UET; Inoue et al., 2012). Instead, we take advantage of the recently developed emotive perspective of employees’ emotional exhaustion, which suggests that the employees’ emotional exhaustion in new ventures is not simply a matter of excessive emotional demand (Wright and Cropanzano, 1998) but that more psychological reasons affect the survival of new ventures (Leiter and Maslach, 2005; Dust et al., 2018).

Past studies have indicated that entrepreneurs‘ empathy, defined as a kind of emotional capacity (e.g., Scott et al., 2010) can induce special emotive feelings in their employees, thus incurring their emotional swings and behaviors (Salovey and Mayer, 1990; Zaki, 2019). If the entrepreneur cannot utilize empathy properly in some circumstances, his/her new ventures may suffer a lot due to employees‘ negative emotions (Huang, 2022). However, it remains invisible how entrepreneurs deal with their empathy, and how they do so affects employees’ emotional exhaustion in new ventures. This connection between empathy and emotional exhaustion is believed to be driven by empathic individuals’ ability to appreciate others’ strain and propensity to put themselves in someone else’s shoes and the generating desire to mitigate it (Bohns, 2016). We argue that how entrepreneurs harness their empathy will have a potential influence on whether their employees will suffer or benefit in emotions.

### Entrepreneurs‘ empathy and employees’ emotional exhaustion

The bulk of literature suggests that empathy should be regarded as a complicated multidimensional construct even though it is an individual-level variable (König et al., 2020), which expresses the “reactions of one individual to the observed experiences of another” (Davis, 1983, p. 113). Specifically, empathy refers to the ability of understanding and sharing other individuals’ emotions and propensities of connecting with and caring about others when individuals are in distress (Davis, 1983; Salovey and Mayer, 1990; Zaki and Ochsner, 2012). Empathy consists of two correlated dimensions, that is, affective and cognitive. The former means sharing similar emotions (i.e., emotional contagion) and having a feeling of sorrow or concern for the other (i.e., empathic concern) while the latter shows the intellectual understanding of another person’s emotions or inner states and relies on perspective-taking (Davis, 1983; Van der Graaff et al., 2017). In this research, we focus on three intra- and interpersonal propensities: emotional contagion (the propensity to “share another person’s emotions at the moment these emotions occur”), empathic concern (the propensity to “respond to another person’s emotions in a given situation without experiencing these emotions”), and perspective-taking (the propensity to “understand the role or point of view of another person, to anticipate the reactions of the other, and to address the other’s perceived needs, motivations, or opinions”) (Wieseke et al., 2012, p. 317).

Emotional exhaustion is a mental state in which negative emotion occurs beyond physical or emotional requirements from one‘s work (Maslach and Jackson, 1984; Leiter and Maslach, 2005). It is a lack of response caused by long-term work pressure, especially work involving high interpersonal contact, which is closely related to the improvement of work and life quality and the performance of organizational functions (Wright and Cropanzano, 1998). Maslach and Jackson (1984) pioneered the study of emotional exhaustion as an early symptom of burnout. When individuals interact with people at work, they are given too much emotional demand but are unable to bear it, which leads to emotional exhaustion due to excessive emotional extension and exhaustion of emotional or psychological resources, which leads to increased withdrawal behaviors, lack of energy, and reduced productivity. When employees in new ventures experience severe emotional exhaustion, their commitment to the organization can be reduced (Li et al., 2016) and their cognitive thinking can deteriorate (Schermuly and Meyer, 2015), resulting in poor work performance. If employees‘ emotional exhaustion does not receive appropriate concern from leaders, the firms can suffer from bad emotions and turnover of the employees (Ladebo, 2009; Van Laethem et al., 2015).

Self-determination theory holds that individuals make free choices about their actions based on a full understanding of individual needs and environmental information (Gagne et al., 1997). This theory emphasizes the role of self in the process of motivation. Self-determinism considers drives, intrinsic needs, and emotions as sources of motivation for self-determined behavior. Individuals tend to have higher levels of performance, health, and well-being if their basic needs (autonomy, competence, and relationships) are met, as opposed to if their basic needs are not. Individuals must continue to meet three basic psychological needs (i.e., autonomy, competence, and relationships) throughout their lives to achieve optimal functioning and experience personal growth and happiness (Gagne and Deci, 2005). This theory recalls the role of entrepreneurs to show care for the employees‘ minds, thoughts, needs, and atmosphere in the workplace, thus causing more positive motivations and reducing negative emotions in employees (Huy and Zott, 2019). How leaders’ cognition, emotion, and psychology influence their followers and organizations has been a major area of focus in micro leadership research (e.g., Dinh et al., 2014). Studies in the utilization of leaders’ cognitive and psychological models (e.g., Mumford et al., 2017) believed that these factors can exert a key influence on employees‘ emotions. When steering the daily operation of the new ventures, entrepreneurs should focus not only on the interfunctional coordination of teams or groups but also on employees‘ potential emotions, which can incur general turmoil or keep the ventures growing steadily (Maitlis and Ozcelik, 2004; Shepherd et al., 2011), as suggested by UET.

Empathy is a significant factor in governing entrepreneur–employee interactions. Even though the nature of empathy is still unclear, research shows concrete consistency in the facilitation of helping behaviors or prosocial behaviors, which can reduce the negative emotions of employees because these behaviors can benefit employees, concern about their needs in the workplace, and settle doubts and distress to satisfy their needs of autonomy, competence, and relationship (Crocetti et al., 2016), thus reducing the feeling of emotional exhaustion. By fostering a more accurate understanding of employees‘ work state and mood, empathy improves the entrepreneurs’ ability to predict employees‘ behavior or reaction (Shepherd et al., 2011). In addition, cooperative behavior is further promoted by adapting one’s behavior to the other’s thoughts and feelings and for the benefit of the other (Homburg et al., 2009). Thus, empathy enhances social interaction by inducing and reinforcing mutually supportive attitudes and behaviors between entrepreneurs and employees (Lazarus, 1991), thus causing more positive emotions and behaviors in employees. Besides, a more empathic entrepreneur will be more thoughtful of the feelings of employees and, thus, more possibly to perceive changes in employees’ facial and vocal expressions, which employees use to hint at distress, strain, or even special needs (Salovey and Mayer, 1990). And when employees express their negative emotions or state of emotional exhaustion, a more empathic entrepreneur will experience similar situations as well, and then he/she will act upon this information. By taking perspectives of employees who are under strain, a highly empathic entrepreneur will be relatively quick in interpreting employees‘ emotional signs and steer them in the right orbit (Tata et al., 2017; De Cock et al., 2020), which can reduce or alleviate the emotional exhaustion by enhancing entrepreneurs’ understanding of employees‘ needs. Therefore, we propose the following hypothesis:

Hypothesis 1: Entrepreneurs‘ empathy has a negative effect on employees’ emotional exhaustion.

### Mediation and moderation roles of psychological empowerment

Psychological empowerment, a sense of control over one‘s work, can be defined as “a process of enhancing feelings of self-efficacy among organizational members through the identification of conditions that foster powerlessness and through their removal by both formal organizational practices and informal techniques of providing efficacy information” (Conger and Kanungo, 1988, p. 474), which can be manifested by four dimensions: meaning, self-determination, competence, and impact (Seibert et al., 2011). To be specific, employees with strong psychological empowerment believe that their work is crucial and significant and fits the goals and beliefs or values (meaning), they own the capability of performing their work successfully (competence), they have a choice to initiate and regulate work activities (self-determination), and they can influence strategic, administrative, or operational outcomes at work (impact) (Spreitzer, 1995). Together, these four cognitions capture an active orientation toward work role and a comprehensive sense of control at work, which has been verified to affect employee outcomes, such as job evolvement, organizational citizenship behavior, job satisfaction, job distress, emotions, and turnover (Avolio et al., 2004; Quiñones et al., 2013).

Upper echelons theory argues that leaders sense and understand the internal and external environment by cognitively processing the information in their daily management, especially sense and experience the emotional information in employees (Huy and Zott, 2019). Therefore, drawing on the upper echelons basis of executives, we believe that entrepreneurs‘ empathy models how employees engage in their work, which influences the four dimensions of psychological empowerment. An entrepreneur with higher empathy has a stronger tendency to perceive and undergo psychological strain experienced by employees, and these negative emotions can cause a reciprocal and conducive dialogue between them (Nahum-Shani et al., 2014) for problem-solving, which can increase a sense of significance or meaning from employees. Besides, an empathic entrepreneur takes perspective to make the employees understand the importance and significance of how they approach their work, which encourages employees to behave properly and effectively to show their values to the organization (Van Laethem et al., 2015). When entrepreneurs convey empathic concern to employees’ work context, emotional swings, and even their work role, employees can sense respect and appreciation and believe that they are an inseparable part of the organization (Avolio et al., 2004), which strengthens their understanding of the impact on the work and reinforce their flexible regulation of work activities (Schermuly and Meyer, 2015). What‘s more, employees voice or speak up for themselves bravely when they hold that entrepreneurs show perspective-taking to consider their negative emotions, thus receiving credit for sharing information with leaders, improving their self-efficacy and confidence in their ability to successfully perform a task, and, in turn, causing effectiveness (Avolio et al., 2004).

In sum, entrepreneurs‘ empathy help employees in new ventures comprehend how to sense the care from firm leaders and engage in their work and behave properly in a way that will be encouraged and rewarded by the entrepreneurs, leading to employees having an incremental feeling of competence and self-determination. In addition, entrepreneurs‘ empathy is conducive to employees to reinforce the meaning and impact of their work. Hence, consistent with previous studies showing that empathy is related to psychological empowerment (Seibert et al., 2011; Sun et al., 2021), we argue that entrepreneurs‘ empathy strengthens employees’ psychological empowerment. However, we also contend that psychological empowerment is a crucial mechanism linking entrepreneurs‘ empathy with employees’ emotional exhaustion.

Employees reduce the state and emotion of exhaustion by adjusting pressure and regulate their emotions and feelings when meeting organizational objectives or requirements (Hobfoll and Shirom, 2001). When viewing their work as meaningful and impactful to new ventures, employees despise the pressure and take initiative to face challenges and setbacks and show their tendency to be persistent in overcoming difficulties and obstacles (Howell et al., 2015). Additionally, employees with a strong sense of self-determination engage in work with great confidence and a sense of freedom in their work and then perform tasks successfully, thus causing a higher sense of achievement (McAllister et al., 2018). Strong personal competence empowers employees themselves with great persistence and confidence to enhance their job satisfaction and organizational commitment (Shepherd et al., 2011), which can alleviate emotional burnout. Importantly, as an active orientation toward work, psychological empowerment can facilitate employees‘ endeavors in shaping their work role, beliefs, and values, and they have great faith in fulfilling every task excellently (Spreitzer, 1995). As discussed earlier, psychologically empowered employees are full of perseverance, resilience, and confidence (Spreitzer, 2008), and they are most likely to engage in more challenging and difficult work with a sense of being master and not the energy that’s about to run out (Leiter and Maslach, 2005; Li et al., 2016), which decreases their emotional exhaustion. Therefore, we propose the following hypothesis:

Hypothesis 2: Psychological empowerment mediates the relationship between entrepreneurs‘ empathy and employees’ emotional exhaustion.

As suggested by self-determination theory, autonomous motivation in the workplace will facilitate employees to experience meaningfulness, competence, self-determination, and impact at work (Spreitzer, 1995; Sun et al., 2021). So psychological empowerment plays a vital role in shaping how external motivational events such as leaders‘ psychology and emotion (i.e., empathy) influence employees‘ emotion fluctuation, for example, emotional exhaustion. Employees regard psychological empowerment as a process of enhancing feelings of self-efficacy (Conger and Kanungo, 1988), which has a crucial effective influence on their emotions. If an employee feels strong empowerment, he or she may have more energy and interest to put themselves into work after being empathically concerned by the entrepreneurs. As a result, entrepreneurs‘ empathy may produce positive emotional feedback for him or her, not emotional exhaustion. Surprisingly, to the best of our knowledge, no research has verified the role of psychological empowerment in the cognitive processes of emotional exhaustion. Our key standpoint is that the reinforcement of psychological empowerment for employees‘ work roles, beliefs, values, and needs may increase their confidence and conserve their psychological resources under awful circumstances and restrain the development of cognitive processes of emotional exhaustion.

Specifically, when highly psychologically empowered employees believe their work is meaningful and have confidence about their ability to do their job, then they produce a sense of control and mastery over work environments (Frieder et al., 2019), which can reduce their feelings of psychological resource exhaustion (Nahum-Shani et al., 2014). When self-determining their work processes and opportunities for independence and freedom, employees can “better highlight their competence and value and feel a greater sense of impact via their self-initiated actions” (Sun et al., 2021, p. 7). They are thus likely to benefit from motivational encouragement that tends to promote positive emotions without perceiving emotional exhaustion. Consequently, when they are given more empathy by leaders, they may develop abundant psychological resources, relaxation and job satisfaction, and expect good expectancies (Scott et al., 2010; Inoue et al., 2012; Zheng et al., 2020). In other words, if empathically concerned or perspective is taken by entrepreneurs, psychologically empowered employees are more prone to make benevolent attributions when unfavorable events occur (Wieseke et al., 2012), thus relieving negative emotions and reducing emotional exhaustion. Hence, for these employees, such empathy from entrepreneurs is more effective in reducing emotional exhaustion.

By contrast, employees who are not psychologically empowered are apt to believe that they are not capable of carrying out a work plan, exerting personal control over the work, and are not significant and meaningful to their work (Walumbwa et al., 2011), which adds more pressure, anxiety, damaged self-esteem, depression, tension, suspicion, and lack of achievement to their work (Pare and Tremblay, 2007), thus causing emotional exhaustion (Ladebo, 2009). Besides, employees who are not psychologically empowered are more likely to produce frustration and tension and contend that they cannot get enough organizational support, thus hurting the feelings of their devotion to the firm (Chatterjee et al., 2021) and performing their tasks badly, which may threaten them with loss of psychological resources and incur emotional exhaustion. In other words, a low level of psychological empowerment may weaken while a high level may strengthen the negative effect of entrepreneurs‘ empathy on employees’ emotional exhaustion. Therefore, we hypothesize the following:

Hypothesis 3: Psychological empowerment moderates the negative relationship between entrepreneurs‘ empathy and employees’ emotional exhaustion such that the negative effects are strengthened as psychological empowerment increases.

## Methodology

### Sample and data collection

We chose China as the research context because it has been experiencing economic transformation and restructuring and showing prosperity in new venture creation (Burt, 2019), especially in the high-tech industry. As a representative of an emerging economy, more high-tech new venture creations in China are not only witnessing the economic vigor but also bringing fierce challenges and pressure for entrepreneurs, who interact with their employees closely to motivate their survival and development (Zhang and Bartol, 2010; Prashantham et al., 2020). Therefore, we decided to collect data from the Yangtze River Delta Area (including three provinces, i.e., Jiangsu, Zhejiang, and Anhui), which is the most developed and prosperous district in China with a very high birth rate of high-tech new ventures.

We conducted a three-wave field study to empirically test the theoretical model and hypotheses about high-tech new ventures, which are defined as firms under 8 years (Zahra and Bogner, 2000). Dyads of employees and entrepreneurs in the sample were surveyed at different times by talking face-to-face to reduce bias. Adopting a temporally segregated design helps reduce potential concerns arising from solely using self-reported and single-source data-collection methods (Tuncdogan et al., 2017). We coded questionnaires numerically on a pair entrepreneur–employee basis.

To increase the response and matching rates between entrepreneurs and employees, we trained members of the research team to understand the connotation of every variable and item to manage the survey well. To ensure the entrepreneur–employee link, entrepreneurs assigned the employees to the respective employees‘ code numbers. The survey lasted 3 months ranging from July to October 2021. At time 1, 151 questionnaires for entrepreneurs (empathy) were issued with numerical code, 1 month later (time 2), 604 questionnaires for employees (psychological empowerment) were issued with paired numerical code, and then another month later (time 3) for emotional exhaustion. The final matched sample consisted of 113 entrepreneurs and 414 employees (total response rate: 69.8%) in 90 new ventures. Of the 414 employees, their average age was 34.77 years (*SD* = 7.18) and 42.27% were female. About 74.64% of them owned a bachelor‘s or above degree with an average organizational tenure of 5.77 years (*SD* = 1.41). Of the 113 entrepreneurs, 36.28% of them had entrepreneurial experiences before they started this new venture and 48.67% owned managerial experience in other firms.

### Measures

All measures were from previous literature, which has been tested in related research many times. Following Brislin‘s (1980) procedure, we invited two bilingual management scholars to translate and back-translate the measurement items to make sure their applicability in the Chinese context. All items were measured by a 5-point Likert scale from 1 (Totally disagree) to 5 (Totally agree), unless otherwise noted.

#### Empathy

We adopted 12 items from McBane (1995) (see Appendix for details) to measure entrepreneurs‘ empathy with minor revision. This scale has three dimensions: perspective-taking (four items, α = 0.83), empathic concern (four items, α = 0.79), and emotional contagion (four items, α = 0.76). The fit indices for the one-factor model were acceptable (χ^2^ = 267.53, df = 85; RMSEA = 0.06; NFI = 0.92; CFI = 0.94; TLI = 0.93).

#### Psychological empowerment

We adopted 12 items from Spreitzer (1995) (see Appendix for details) to measure psychological empowerment. This scale has four dimensions: meaning (three items, α = 0.87), competence (three items, α = 0.78), self-determination (three items, α = 0.81), and impact (three items, α = 0.85). These 12 items were averaged to create one score for each individual. The fit indices for the one-factor model were acceptable (χ^2^ = 783.67, df = 346; RMSEA = 0.05; NFI = 0.97; CFI = 0.96; TLI = 0.91). The overall reliability coefficient was 0.85. In this study, we conceptualized psychological empowerment as a group-level construct, it is necessary to aggregate individual perceptions of psychological empowerment to the group level. Therefore, we computed the median value of within-group agreement γwg and the intraclass correlation coefficients (ICC1 and ICC2). The median γwg is 0.91, and ICC(1) = 0.42, ICC(2) = 0.86, *F* = 9.47, *p* < 0.01, all supporting the aggregation (Bliese, 2000).

#### Emotional exhaustion

A 9-item scale developed by Maslach and Jackson (1981) (see Appendix for details) was used to measure emotional exhaustion. The reliability coefficient was 0.84. Because we conceptualized emotional exhaustion as a group-level construct, we aggregated employees’ ratings to the group level. The median of emotional exhaustion γwg is 0.90 and ICC(1) = 0.50, ICC(2) = 0.82, *F* = 8.53, *p* < 0.001, thus supporting the aggregation.

#### Controls

We controlled for employees‘ age, gender (1 = male, 0 = female), education (1 = bachelor or above, 0 = below bachelor), and tenure (years worked with the organization) because these factors reflect the level of experience and knowledge and their emotional conditions when employees face setbacks (Zhang et al., 2010; Seibert et al., 2011). Further, we also controlled for entrepreneurs‘ managerial experience (1 = yes, 0 = no) which can influence how they behave in their daily management, especially how they can affect employees‘ psychology and emotions when they are under strain (Van Laethem et al., 2015). Finally, since the entrepreneurial experience can influence entrepreneurs‘ empathy of how and when to show to employees (Toledano, 2020), we created a dummy variable to control for entrepreneurial experience (1 = yes, 0 = no).

## Results

### Data quality analyses

Table 1 presents the correlation matrix of all variables in the study. Before hypothesis testing, we carried out the confirmatory factor analyses (CFA) to assess the validity of the measurements as shown in Table 2. After comparing the five models, we found that hypothesized three-factor model (model 1) had the best indices of fitness: χ2 = 813.52, NFI (normed fit index) = 0.94, CFI (comparative fit index) = 0.97, TLI (Tucker-Lewis index) = 0.91, and RMSEA (root mean squared error of approximation) = 0.05. The changes in Δχ2 and degree of freedom (d.f.) of other models showed significant differences compared with model 1 (*p* < 0.01) and their indices of fitness were worse than the baseline model. Therefore, we continued with subsequent analyses by treating the three measures as disparate constructs.

**TABLE 1:** Correlation matrix of all variables.

Variables	1	2	3	4	5	6	7	8	9
1. Age	−								
2. Gender	0.04	−							
3. Education	0.03	0.10	−						
4. Tenure	0.09	0.05	–0.08	−					
5. Managerial experience	0.04	0.01	0.01	0.11	−				
6. Entrepreneurial experience	0.07	0.03	0.10	–0.06	0.09	−			
7. Empathy	−.00.0.02	−.00.0.12	0.08	0.03	0.01	0.13	***0.56***		
8. Psychological empowerment	0.04	0.07	0.09	0.08	–0.04	0.20*	0.29**	***0.52***	
9. Emotional exhaustion	−0.19*	0.01	0.02	–0.02	0.03	0.06	0.41***	0.33***	***0.57***
Mean	34.77	0.58	0.75	5.77	0.49	0.36	3.69	3.91	3.82
SD	5.18	0.14	0.21	1.41	0.16	0.10	0.87	0.74	0.80

AVE (average variance extracted) is listed in bold italic along the diagonal. *p <0.05; **p <0.01; ***p <0.001.

**TABLE 2:** The results of confirmatory factor analyses.

Model	Description	χ^2^	df	NFI	CFI	TLI	RMSEA	Δχ^2^(d.f.)
1	Hypothesized three-factor model	813.52	361	0.94	0.97	0.91	0.05	Baseline
2	Two-factor model (empathy and emotional exhaustion combined)	1598.47	366	0.84	0.81	0.79	0.13	784.95(5)**
3	Two-factor model (psychological empowerment and empathy combined)	1654.39	376	0.80	0.74	0.72	0.14	840.89(15)**
4	Two-factor model (psychological empowerment and emotional exhaustion combined)	1933.72	379	0.73	0.70	0.67	0.16	1120.20(18)**
5	One-factor model (three variables combined)	2439.14	384	0.67	0.63	0.64	0.17	1625.62(23)**

**p < 0.01. All alternative models were compared with the baseline model.

### Hypotheses testing

As suggested by Bliese (2000), the HLM approach is appropriate for testing multilevel data as shown in this study. HLM can account for the possible non-independence of observations since employees are nested within entrepreneurs (i.e., 414 employees reporting to 113 entrepreneurs). To test the hypotheses, we used HLM analyses to account for the nested structure of the data (i.e., 414 employees reporting to 113 entrepreneurs) using HLM 6.0. Indeed, the ICC1 was 0.42 for psychological empowerment and 0.50 for emotional exhaustion, thus supporting the suitableness of this approach.

Hypothesis 1 argues that entrepreneurs‘ empathy has a negative effect on employees’ emotional exhaustion. As shown in Table 3 (Model 2), entrepreneurs‘ empathy was negatively related to employees’ emotional exhaustion (β = –0.14, *p* < 0.05), thus supporting Hypothesis 1. Hypothesis 2 states that psychological empowerment mediates the relationship between entrepreneurs‘ empathy and employees’ emotional exhaustion. The results in models 1 and 2 showed that psychological empowerment partially mediates the above relationship, thus giving support to Hypothesis 2. Finally, the results in model 3 expressed support for Hypothesis 3, which argues that psychological empowerment significantly moderates the negative relationship between entrepreneurs‘ empathy and employees’ emotional exhaustion (β = 0.25, *p* < 0.01). In all, three hypotheses were supported.

**TABLE 3:** The results of hypotheses testing by using HLM.

Variables	Model 1:	Model 2:	Model 3:
	Psychological empowerment	emotional exhaustion	emotional exhaustion
Age	0.04(0.03)	0.02(0.04)	0.03(0.04)
Gender	0.12(0.13)	0.17(0.13)	0.19(0.14)
Education	0.06(0.11)	0.09(0.11)	0.07(0.11)
Tenure	0.01(0.07)	0.14(0.09)	0.13(0.09)
Managerial experience	−0.04(0.04)	0.10(0.07)	0.09(0.07)
Entrepreneurial experience	0.03(0.05)	0.10(0.06)*	0.11(0.06)*
Empathy	0.19(0.07)**	−0.14(0.07)*	−0.16(0.07)*
Psychological empowerment		−0.29(0.11)**	−0.23(0.11)*
Empathy * Psychological empowerment			0.25(0.09)**
Variance (level 2 intercept)	0.214	0.177	0.150
Variance (level 1 residual)	0.714	0.653	0.607
Pseudo R2	0.213	0.237	0.254
Model deviance	733.62	827.24	856.93

*p < 0.05, **p <0.01. Unstandardized coefficients with standard errors in parentheses.

**FIGURE 2:**
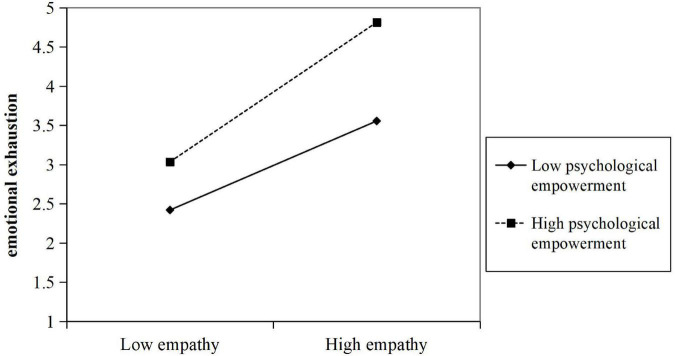
Two-way interaction effect.

In line with the results, we plotted the two-way interaction effect in Figure 2 based on Aiken and West‘s (1991) procedures. Obviously as indicated in Figure 2, when employees are psychologically empowered at a high level, entrepreneurs‘ empathy had a stronger negative effect on employees’ emotional exhaustion, suggesting that psychological empowerment could reinforce the function of empathy in alleviating employees’ emotional exhaustion and encouraging their persistence and resilience in the workplace.

### *Post hoc* analysis

To perform *post hoc* analysis, we tested the effects of sub-dimensions of empathy (emotional contagion, empathic concern, and perspective-taking) to confirm whether one dimension is more influential than the other two in affecting psychological empowerment, and subsequently employees’ emotional exhaustion. We re-ran the multilevel analyses by using HLM for the three dimensions of empathy and got similar results for each dimension. The significance and the pattern remain the same for all dimensions.

## Discussion and implication

Although the UET stresses the pertinence of leaders‘ empathy in leader–employee interactions (Shepherd et al., 2011; Huy and Zott, 2019), it is still limited about the effect of empathy on the emotional swings in employees. Besides, self-determination theory suggests that employees’ emotions may largely be affected by leaders‘ cognitive donation of the feelings to motivate the active orientation of meaning, competence, self-determination, and impact of work (Spreitzer, 2008), but its influential mechanism is ambiguous in new ventures. Hence, to extend this emerging scholarly conversation, we argued and tested how and when entrepreneurs‘ empathy shows an effect on employees’ emotional exhaustion. Through a multilevel analysis, we found that entrepreneurs‘ empathy attenuates employees’ emotional exhaustion while psychological empowerment partially mediates the above relationship. Most interestingly, psychological empowerment moderates the relationship between entrepreneurs‘ empathy and employees’ emotional exhaustion significantly, that is to say, when employees are highly psychologically empowered, the negative effect of entrepreneurs‘ empathy on employees’ emotional exhaustion is strengthened. These findings have noteworthy theoretical and practical implications.

Theoretical Implications: First, by investigating the consequences of entrepreneurs’ trait of empathy for emotional fluctuation, we advance the emotion-focused upper echelons perspective (Hambrick, 2007) to study employees’ emotional exhaustion. Especially, we respond to recent calls from scholars that, instead of concentrating on the consequences of observable characteristics of leaders in new ventures (e.g., Tata et al., 2017; Sharifi-Tehrani et al., 2022), address the more complicated aspects of leaders in new venture context. By emphasizing entrepreneurs’ empathy, we also develop a new perspective for understanding whether new ventures are transient in their management of emotions. Besides, previous studies have lost sight of the dyadic nature of emotion and psychological interaction and mainly focused on leaders‘ empathy as an essential predictor of employees’ satisfaction (Dust et al., 2018; Cameron et al., 2022). Indeed, entrepreneurs‘ empathy has long been regarded as an important driver of employees‘ needs and generate favorable outcome such as job satisfaction (Zaki and Ochsner, 2012). Drawing from the emotion-focused upper echelons perspective, we develop an entrepreneur–employee empathy model and demonstrate how entrepreneurs‘ empathy help translate employee emotion into intangible resources. Our model is consistent with and enhances the understanding of prior studies.

Second, drawing upon self-determination theory, we verify the mediating role of psychological empowerment. This finding answered the call to investigate specific functions of intrinsic motivation (Gagne and Deci, 2005). The current study examines that psychological empowerment (a kind of autonomous motivation) links empathy and emotional exhaustion by partially mediating their relationship, thus going beyond the self-determination theory as the latent mechanism of the psychological process. Moreover, the findings depict a full view of the trickle-down effect of psychological empowerment on employees’ emotional exhaustion in new ventures. Most importantly, we discuss the specific feature of entrepreneurs’ empathy and employees‘ emotions in the context of new ventures by drawing on upper echelons’ perspectives and self-determination theory, which can facilitate cross-fertilization in theories.

Third, we investigate the moderating role of psychological empowerment and extend a deep understanding of the boundary conditions of empathy by testifying that psychological empowerment that reinforces employees’ cognitive functioning, has a strengthening effect (Sun et al., 2012) on the influence of entrepreneurs’ empathy. Prior empathy research mainly focuses on the idea that psychological displays by entrepreneurs can generate corresponding changes in employees’ affective states (Vachon and Lynam, 2016), but the question of “when” remain unsolved. By adopting an employee-centric perspective of boundary conditions, the current research offsets this defect.

### Practical implications

Our research findings have important implications for entrepreneurs and employees. For entrepreneurs, they should consider employees‘ emotions in the context of new ventures, because these emotions affect their consequent behavior, which may be harmful to the new ventures if poorly handled. Particularly in times of VUCA (volatility, uncertainty, complexity, and ambiguity) and when entrepreneurs sense distress and failure in employees, “walking in the shoes of employees” can create helpful experiences enabling them to develop a better skill for empathy (Allemand et al., 2015) and develop mutual respect and trust with employees (Spreitzer, 2008). Empathic entrepreneurs are likely to carry out empowerment practices to strengthen the autonomous work atmosphere, to better communicate the vision of the new venture with their employees, and stimulate resilience and confidence among employees, thus causing them to perform tasks successfully.

For employees, they should maintain a better sense of psychological empowerment to perceive their work role well. When facing setbacks or negative emotions, they should ask entrepreneurs for help bravely and communicate with them to deal with bad emotions, and reduce the tendency of exhaustion. It is therefore vitally important for employees to learn how to effectively regulate their state and psychology of emotional exhaustion, steering positive consequences of empathy. Nevertheless, it is also crucial to emphasize our finding that without strong psychological empowerment, engaging in high levels of emotional exhaustion can lower the effect of empathy reversely.

### Limitations and future directions

This study owns several strengths, but it still has several limitations that should be addressed for future research. First, despite using time-lagged data, we still cannot conclude that there is causality among entrepreneurs‘ empathy, psychological empowerment, and employees’ emotional exhaustion, which is usual in field studies like this one, so it is untenable for only one test. Therefore, there is a need to obtain more time-lagged data to further study the above relationships. Additionally, because individuals, especially those with ample experience, can influence the extent to which they feel empathic (Bohns, 2016), it is necessary to employ experimental research designs to judge the causality of the model.

Second, the data of our study are mainly from the Yangtze River Delta Area (including three provinces, i.e., Jiangsu, Zhejiang, and Anhui), one of the most developed regions in China, and focuses on high-tech new ventures, which can harm the generalizability of the results. Future research can contribute to the generalizability of this study by adopting data from multi-regions and multi-industries. Most importantly, attention should also be paid to comparing findings resulting from different regions and industries to enrich the conceptual model.

Third, given the samples, entrepreneurs gauge their performance in empathy while employees evaluate their understandings of psychological empowerment and emotional exhaustion, which may cause subjective bias. If possible, in future, research scholars can change the evaluation methods, that is, they can ask employees to gauge the performance of entrepreneurs‘ empathy, which may be more objective compared with entrepreneurs‘ self-reported empathy.

## Conclusion

This study contributes to a deep understanding of the motivational impact of entrepreneurs‘ empathy on employees’ emotional exhaustion. Empirical results indicate that entrepreneurs‘ empathy can model employees‘ emotions and reduce their emotional exhaustion through a high level of psychological empowerment. However, the enhanced efforts of entrepreneurs‘ empathy are only conducive to the alleviation of emotional exhaustion when employees are highly psychologically empowered.

## Data availability statement

The original contributions presented in the study are included in the article/Supplementary material, further inquiries can be directed to the corresponding author/s.

## Author contributions

JX worked on the discussion part and revision. BD worked for modeling and empirical study. YW contributed to introduction and revision. NJ worked for hypotheses and revision. YG contributed to the introduction and hypotheses. All authors contributed to the article and approved the submitted version.

## References

[B1] Aiken L. S. West S. G. (1991). *Multiple regression: Testing and Interpreting Interactions.* Newbury Park, CA: Sage.

[B2] Allemand M. Steiger A. E. Fend H. A. (2015). Empathy development in adolescence predicts social competencies in adulthood. *J. Pers.* 83 229–241. 10.1111/jopy.12098 24684661

[B3] Antonakis J. Ashkanasy N. M. Dasborough M. T. (2009). Does leadership need emotional intelligence? *Leadersh. Q.* 20 247–261. 10.1016/j.leaqua.2009.01.006

[B4] Avolio B. J. Zhu W. Koh W. Bhatia P. (2004). Transformational leadership and organizational commitment: mediating role of psychological empowerment and moderating role of structural distance. *J. Organ. Behav.* 25 951–968. 10.1002/job.283

[B5] Barney J. B. (2001). Is the resource-based “view” a useful perspective for strategic management research? Yes. *Acad. Manage. Rev.* 26 41–56. 10.2307/259393

[B6] Bliese P. D. (2000). “Within-group agreement non-independence, and reliability,” in *Multilevel Theory, Research, and Methods in Organizations: Foundations, Extensions, and New Directions*, eds Klein K. J. Kozloski S. W. J. (San Francisco, CA: Jossey-Bass), 349–381.

[B7] Bloom P. (2017). Empathy and its discontents. *Trends Cogn. Sci.* 21 24–31. 10.1016/j.tics.2016.11.004 27916513

[B8] Bohns V. (2016). (Mis)understanding our influence over others: a review of the underestimation-of-compliance effect. *Curr. Dir. Psychol.* 25 119–123. 10.1177/0963721415628011

[B9] Brislin R. W. (1980). “Translation and content analysis of oral and written materials,” in *Handbook of Cross-Cultural Psychology: Methodology*, eds Triandis H. C. Berry J. W. (Boston, MA: Allyn and Bacon), 389–444. 10.3390/healthcare6030093

[B10] Brown M. E. Treviño L. K. Harrison D. A. (2005). Ethical leadership: a social learning perspective for construct development and testing. *Organ. Behav. Hum. Decis. Process.* 97 117–134. 10.1016/j.obhdp.2005.03.002

[B11] Burt R. S. (2019). Network disadvantaged entrepreneurs: density, hierarchy, and success in china and the west. *Entrep. Theory Pract.* 43 19–50. 10.1177/1042258718783514

[B12] Calvert S. L. Strouse G. A. Murray K. J. (2006). Empathy for adolescent’s role model selection and learning of DVD content. *J. Appl. Dev. Psychol.* 27 444–455. 10.1016/j.appdev.2006.06.005

[B13] Cameron C. D. Conway P. Scheffer J. A. (2022). Empathy regulation, prosociality, and moral judgment. *Curr. Opin. Psychol.* 44 188–195. 10.1016/j.copsyc.2021.09.011 34695643

[B14] Carpenter M. A. Geletkanycz M. A. Sanders W. G. (2004). Upper echelons research revisited: antecedents, elements, and consequences of top management team composition. *J. Manage.* 30 749–778. 10.1016/j.jm.2004.06.001

[B15] Chatterjee I. Cornelissen J. Wincent J. (2021). Social entrepreneurship and values work: the role of practices in shaping values and negotiating change. *J. Bus. Ventur.* 36:106064. 10.1016/j.jbusvent.2020.106064

[B16] Conger J. A. Kanungo R. N. (1988). The empowerment process: integrating theory and practice. *Acad. Manage. Rev.* 13 471–482. 10.2307/258093

[B17] Cooley E. Payne B. K. Cipolli W. III Cameron C. D. Berger A. Gray K. (2017). The paradox of group mind: “people in a group” have more mind than “a group of people”. *J. Exp. Psychol. Gen.* 146:691. 10.1037/xge0000293 28368192

[B18] Crocetti E. Moscatelli S. Van der Graaff J. Rubini M. Meeus W. Branje S. (2016). The interplay of self-certainty and prosocial development in the transition from late adolescence to emerging adulthood. *Eur. J. Personal.* 30 594–607. 10.1002/per.2084

[B19] Cropanzano R. Rupp D. E. Byrne Z. S. (2003). The relationship of emotional exhaustion to work attitudes, job performance, and organizational citizenship behaviors. *J. Appl. Psychol.* 88 160–169. 10.1037/0021-9010.88.1.160 12675403

[B20] Davis M. H. (1983). Measuring individual differences in empathy: evidence for a multidimensional approach. *J. Pers. Soc. Psychol.* 44 113–126. 10.1037/0022-3514.44.1.113

[B21] De Cock R. Denoo L. Clarysse B. (2020). Surviving the emotional rollercoaster called entrepreneurship: therole of emotion regulation. *J. Bus. Ventur.* 35. 10.1016/j.jbusvent.2019.04.004

[B22] Decety J. Bartal I. B. A. Uzefovsky F. Knafo-Noam A. (2016). Empathy as a driver of prosocial behavior: highly conserved neurobehavioural mechanisms across species. *Philos. Trans. R. Soc. B Biol. Sci.* 371:1686. 10.1098/rstb.2015.0077 26644596 PMC4685523

[B23] Dinh J. E. Lord R. G. Gardner W. L. Meuser J. D. Liden R. C. Hu J. (2014). Leadership theory and research in the new millennium: current theoretical trends and changing perspectives. *Leadersh. Q* 25 36–62. 10.1016/j.leaqua.2013.11.005

[B24] Dong B. Xu H. Luo J. Nicol C. D. Liu W. (2020). Many roads lead to Rome: how entrepreneurial orientation and trust boost the positive network range and entrepreneurial performance relationship. *Ind. Mark. Manag.* 88 173–185. 10.1016/j.indmarman.2020.04.016

[B25] Dust B. Resickb J. Margolisc A. Mawritzb B. Greenbaum L. (2018). Ethical leadership and employee success: examining the roles of psychological empowerment and emotional exhaustion. *Leadersh. Q.* 29 570–583. 10.1016/j.leaqua.2018.02.002

[B26] Eklund J. H. (2006). Personality and social sciences-empathy and viewing the other as a subject. *Scand. J. Psychol.* 47 399–409. 10.1111/j.1467-9450.2006.00521.x 16987209

[B27] Fiori M. Ortony A. (2016). Are emotionally intelligent individuals hypersensitive to emotions? Testing the curse of emotion. *Acad. Manag. Proc.* 2016:10023. 10.5465/AMBPP.2016.10023abstract

[B28] Frieder R. E. Ferris G. R. Perrewé P. L. Wihler A. Brooks C. D. (2019). Extending the metatheoretical framework of social/political influence to leadership: political skill effects on situational appraisals, responses, and evaluations by others. *Pers. Psychol.* 72 543–569. 10.1111/peps.12336

[B29] Gagne M. Deci E. L. (2005). Self-determination theory and work motivation. *J. Organ. Behav.* 26 331–362. 10.1002/job.322

[B30] Gagne M. Senecal C. B. Koestner R. (1997). Proximal job characteristics, feelings of empowerment, and intrinsic motivation: a multidimensional model. *J. Appl. Soc. Psychol.* 27 1222–1240. 10.1111/j.1559-1816.1997.tb01803.x

[B31] Halbesleben J. R. Bowler W. M. (2007). Emotional exhaustion and job performance: the mediating role of motivation. *J. Appl. Psychol.* 92 93–106. 10.1037/0021-9010.92.1.93 17227154

[B32] Hambrick D. C. (2007). Upper echelons theory: an update. *Acad. Manage. Rev.* 32 334–343. 10.2307/20159303

[B33] Hambrick D. C. Lovelace J. B. (2018). The role of executive symbolism in advancing new strategic themes in organizations: a social influence perspective. *Acad. Manage. Rev.* 43 110–131. 10.5465/amr.2015.0190

[B34] Hambrick D. C. Mason P. (1984). Upper echelons: the organization as a reflection of its top managers. *Acad. Manage. Rev.* 9 193–206. 10.5465/amr.1984.4277628

[B35] Hill N. S. Kang J. H. Seo M. G. (2014). The interactive effect of leader-member exchange and electronic communication on employee psychological empowerment and work outcomes. *Leadersh. Q.* 25 772–783. 10.1016/j.leaqua.2014.04.006

[B36] Hobfoll S. E. Shirom A. (2001). “Conservation of resources theory: applications to stress and management in the workplace,” in *Handbook of Organizational Behavior*, ed. Golembiewski R. T. (New York, NY: Marcel Dekker), 57–80.

[B37] Homburg C. Wieseke J. Bornemann T. (2009). Implementing the marketing concept at the employee-customer interface: the role of customer need knowledge. *J. Mark.* 73 64–81. 10.1509/jmkg.73.4.64 11670861

[B38] Howell T. M. Harrison D. A. Burris E. R. Detert J. R. (2015). Who gets credit for input? demographic and structural status cues in voice recognition. *J. Appl. Psychol.* 100 1765–1784. 10.1037/apl0000025 25915784

[B39] Huang Y. (2022). Spiritual leadership and job engagement: the mediating role of emotion regulation. *Front. Psychol.* 13:844991. 10.3389/fpsyg.2022.844991 35496230 PMC9046577

[B40] Huy Q. Zott C. (2019). Exploring the affective underpinnings of dynamic managerial capabilities: how managers’ emotion regulation behaviors mobilize resources for their firms. *Strateg. Manage. J.* 40 28–54. 10.1002/smj.2971

[B41] Inoue A. Kawakami N. Tsuno K. Shimazu A. Tomioka K. Nakanishi M. (2012). Job demands, job resources, and work engagement of Japanese employees: a prospective cohort study. *Int. Arch. Occup. Environ. Health* 86 441–449. 10.1007/s00420-012-0777-1 22562520

[B42] Kim H. Richardson S. L. (2003). Motion picture impacts on destination images. *Ann. Touris. Res.* 30 216–237. 10.1016/S0160-7383(02)00062-2

[B43] König A. Graf-Vlachy L. Bundy J. Little L. M. (2020). A blessing and a curse: how ceos’ trait empathy affects their management of organizational crises. *Acad. Manage. Rev.* 45 130–153. 10.5465/amr.2017.0387

[B44] Ladebo O. J. (2009). Emotional exhaustion and strain reactions: perceived organizational support as a moderator. *South Afr. J. Psychol.* 39 46–58. 10.1177/008124630903900104

[B45] Lazarus R. S. (1991). *Emotion and Adaptation.* New York, NY: Oxford University Press.

[B46] Leiter M. P. Maslach C. (2005). “A mediation model of job burnout,” in *Handbook of Research companion to Organizational Health Psychology*, eds Antoniou A. S. G. Cooper C. L. (Cheltenham: Edward Elgar), 544–564.

[B47] Levy J. (1997). A note on empathy. *New Ideas Psychol.* 15 179–184. 10.1016/s0732-118x(97)00007-x

[B48] Li J. Lee T. W. Mitchell T. R. Hom P. W. Griffeth R. W. (2016). The effects of proximal withdrawal states on job attitudes, job searching, intent to leave, and employee turnover. *J. Appl. Psychol.* 101 1436–1456. 10.1037/apl0000147 27504652

[B49] Maitlis S. Ozcelik H. (2004). Toxic decision processes: a study of emotion and organizational decision making. *Organ. Sci.* 15 375–393. 10.1287/orsc.1040.0070 19642375

[B50] Maslach C. Jackson S. E. (1981). The measurement of experienced burnout. *J. Organ. Behav.* 2 99–113. 10.1002/job.4030020205

[B51] Maslach C. Jackson S. E. (1984). Burnout in organizational settings. *Appl. Soc. Psychol. Annu.* 5 133–153.

[B52] McAllister C. P. Ellen B. P. Ferris G. R. (2018). Social influence opportunity recognition, evaluation, and capitalization: increased theoretical specification through political Skill’s dimensional dynamics. *J. Manage.* 44 1926–1952. 10.1177/0149206316633747

[B53] McBane D. A. (1995). Empathy and the salesperson: a multidimensional perspective. *Psychol. Mark.* 12 349–370. 10.1002/mar.4220120409

[B54] Mumford M. D. Todd E. M. Higgs C. McIntosh T. (2017). Cognitive skills and leadership performance: the nine critical skills. *Leadersh. Q.* 28 24–39. 10.1016/j.leaqua.2016.10.012

[B55] Nahum-Shani I. Henderson M. M. Lim S. Vinokur A. D. (2014). Supervisor support: does supervisor support buffer or exacerbate the adverse effects of supervisor undermining? *J. Appl. Psychol.* 99 484–503. 10.1037/a0035313 24490969 PMC7433003

[B56] Packard M. Burnham T. (2021). Do we understand each other? Toward a simulated empathy theory for entrepreneurship. *J. Bus. Vent.* 36:106076. 10.1016/j.jbusvent.2020.106076

[B57] Pare G. Tremblay M. (2007). The influence of high-involvement human resources practices, procedural justice, organizational commitment, and citizenship behaviors on information technology professionals’ turnover intentions. *Group Organ. Manage.* 32 326–357. 10.1177/1059601106286875

[B58] Phillips J. G. Reddie L. (2007). Decisional style and self-reported Email use in the workplace. *Comput. Hum. Behav.* 23, 2414–2428. 10.1016/j.chb.2006.03.016

[B59] Prashantham S. Zhou A. J. Dhanaraj C. (2020). Depth vs. breadth: network strategy in emerging markets. *Manage. Organ. Rev.* 16 229–260. 10.1017/mor.2019.54

[B60] Quiñones M. den Broeck A. V. De Witte H. (2013). Do job resources affect work engagement via psychological empowerment? A mediation analysis. *Rev. Psicol. Trab. Organ.* 29 127–134. 10.5093/tr2013a18

[B61] Salovey P. Mayer J. D. (1990). Emotional intelligence. *Imag. Cogn. Pers.* 9 185–211. 10.2190/DUGG-P24E-52WK-6CDG 22612255

[B62] Schermuly C. C. Meyer B. (2015). Good relationships at work: the effects of leader–member exchange and team-member exchange on psychological empowerment, emotional exhaustion, and depression. *J. Organ. Behav.* 37 673–691. 10.1002/job.2060

[B63] Scott B. A. Colquitt J. A. Paddock E. L. Judge T. A. (2010). A daily investigation of the role of manager empathy on employee well-being. *Organ. Behav. Hum. Decis. Process.* 113 127–140. 10.1016/j.obhdp.2010.08.001

[B64] Seibert S. E. Wang G. Courtright S. H. (2011). Antecedents and consequences of psychological and team empowerment in organizations: a meta-analytic review. *J. Appl. Psychol.* 96 981–1003. 10.1037/a0022676 21443317

[B65] Sharifi-Tehrani M. Seyfi S. Zaman M. (2022). At the intersection of tourism social entrepreneurship and empathy: development and validation of an empathy scale. *J. Bus. Res.* 141 433–447. 10.1016/j.jbusres.2021.11.041

[B66] Shepherd D. A. Patzelt H. Wolfe M. (2011). Moving forward from project failure: negative emotions, affective commitment, and learning from the experience. *Acad. Manage. J.* 54 1229–1259. 10.5465/amj.2010.0102

[B67] Spreitzer G. M. (1995). Psychological empowerment in the workplace: dimensions, measurement, and validation. *Acad. Manage. J.* 38 1442–1465. 10.2307/256865

[B68] Spreitzer G. M. (2008). “Taking stock: a review of more than twenty years of research on empowerment at work,” in *The Sage Handbook of Organizational Behavior*, eds Cooper C. Barling J. (Thousand Oaks, CA: Sage Publications), 54–73.

[B69] Stock R. M. Bednarek M. (2014). As they sow, so shall they reap: customers’ influence on customer satisfaction at the customer interface. *J. Acad. Mark. Sci.* 42 400–414. 10.1007/s11747-013-0355-4

[B70] Sun L. Y. Zhang Z. Qi J. Chen Z. X. (2012). Empowerment and creativity: a cross-level investigation. *Leadersh. Q.* 23 55–65. 10.1016/j.leaqua.2011.11.005

[B71] Sun S. Burke M. Chen H. Tan Y. Zhang J. Hou L. (2021). Mitigating the psychologically detrimental effects of supervisor undermining: joint effects of voice and political skill. *Hum. Relat.* 75 87–112. 10.1177/0018726721992849

[B72] Tang S. Gray K. (2021). Feeling empathy for organizations: moral consequences, mechanisms, and the power of framing. *J. Exp. Soc. Psychol.* 96:104147. 10.1016/j.jesp.2021.104147

[B73] Tata A. Martinez D. L. Garcia D. Oesch A. Brusoni S. (2017). The psycholinguistics of entrepreneurship. *J. Bus. Ventur. Insights* 7 38–44. 10.1016/j.jbvi.2017.02.001

[B74] Toledano N. (2020). Promoting ethical reflection in the teaching of social entrepreneurship: a proposal using religious parables. *J. Bus. Ethics.* 164 115–132. 10.1007/s10551-018-4077-x

[B75] Tuncdogan A. Acar O. A. Stam D. (2017). Individual differences as antecedents of leader behavior: towards an understanding of multi-level outcomes. *Leadersh. Q.* 28 40–64. 10.1016/j.leaqua.2016.10.011

[B76] Vachon D. D. Lynam D. R. (2016). Fixing the problem with empathy: development and validation of the affective and cognitive measure of empathy. *Assessment* 23 135–149. 10.1177/1073191114567941 25612628

[B77] Van der Graaff J. Carlo G. Crocetti E. Koot H. M. Branje S. (2017). Prosocial behavior in adolescence: gender differences in development and links with empathy. *J. Youth Adolesc.* 47 1086–1099. 10.1007/s10964-017-0786-1 29185207 PMC5878203

[B78] Van Laethem M. Beckers D. G. Kompier M. A. Kecklund G. van den Bossche S. N. Geurts S. A. (2015). Bidirectional relations between work-related stress, sleep quality and perseverative cognition. *J. Psychosomat. Res.* 79 391–398. 10.1016/j.jpsychores.2015.08.011 26526314

[B79] Walumbwa F. O. Mayer D. M. Wang P. Wang H. Workman K. Christensen A. L. (2011). Linking ethical leadership to employee performance: the roles of leader-member exchange, self-efficacy, and organizational identification. *Organ. Behav. Hum. Decis. Process.* 115 204–213. 10.1016/j.obhdp.2010.11.002

[B80] Wieseke J. Geigenmuller A. Kraus F. (2012). On the role of empathy in customer employee interactions. *J. Serv. Res.* 15 316–331. 10.1177/1094670512439743

[B81] Wright T. A. Cropanzano R. (1998). Emotional exhaustion as a predictor of job performance and voluntary turnover. *J. Appl. Psychol.* 83 486–493. 10.1037/0021-9010.83.3.486 9648526

[B82] Zahra S. A. Bogner W. C. (2000). Technology strategy and software new ventures’ performance: exploring the moderating effect of the competitive environment. *J. Bus. Ventur.* 15 135–173. 10.1016/S0883-9026(98)00009-3

[B83] Zaki J. (2019). Integrating empathy and interpersonal emotion regulation. *Annu. Rev. Psychol.* 71 517–540. 10.1146/annurev-psych-010419-050830 31553672

[B84] Zaki J. Ochsner K. (2012). The neuroscience of empathy: progress, pitfalls and promise. *Nat. Neurosci.* 15 675–680. 10.1038/nn.3085 22504346

[B85] Zhang X. M. Bartol K. M. (2010). Linking empowering leadership and employee creativity: the influence of psychological empowerment, intrinsic motivation, and creativity process engagement. *Acad. Manage. J.* 53 107–128. 10.5465/amj.2010.48037118

[B86] Zheng C. C. Ahsan M. DeNoble A. F. (2020). Entrepreneurial networking during early stages of opportunity exploitation: agency of novice and experienced new venture leaders. *Entrep. Theory Pract.* 44 671–699. 10.1177/1042258719844715

