# Supplementary material for: A whirlpool of emotion: How entrepreneurs’ empathy affects employees‘ emotional exhaustion
Source: Front Psychol. 2022 Aug 8;13:933628. doi: 10.3389/fpsyg.2022.933628 (PMC9395146; doi:10.3389/fpsyg.2022.933628)
Supplement: Supplementary file 1 [file Data_Sheet_1.pdf]

APPENDIX

Scale items for construct measures

Empathy (revised accordingly) (5-point scale: totally disagree to totally agree)

Perspective-Taking

1. I try to look at the employees' side of a disagreement before I make a decision.
2. When I am upset at employees, I usually try to put myself in their shoes."
3. I believe that there are two sides to every question and try to look at them both.

Empathic Concern

1. I would describe myself as a pretty soft-hearted person.
2. If an employee is unhappy, I quickly realize this, even if I do not know the person well
3. Employees' misfortunes usually disturb me a great deal.
4. I often have tender concerned feelings for employees less fortunate than me.

Emotional Contagion

1. I become nervous if employees around me are nervous in my firm
2. I cannot remain cool if employees around me are excited.
3. I get easily infected by the mood of employees.
4. If I see unhappy employees, I feel unhappy myself
5. If I see happy employees, I feel happy myself

Source: McBane, D. A, 1995. Empathy and the salesperson: a multidimensional perspective. Psychol. Market. 12(4), 349-370.

Psychological Empowerment (5-point scale: totally disagree to totally agree)

Meaning

1. The work I do is very important to me(meaning 1).
2. My job activities are personally meaningful to me(meaning 2)
3. The work I do is meaningful to me(meaning 3)

Competence

1. I am confident about my ability to do my job(competence 1).
2. I am self-assured about my capabilities to perform my work activities(competence 2).
3. I have mastered the skills necessary for my job(competence 3)

Self-Determination

1. I have significant autonomy in determining how I do my job(self-determination 1).
2. I can decide on my own how to go about doing my work(self-determination 2).
3. I have considerable opportunity for independence and freedom in how I do my job(self-determination 3).

Impact

1. My impact on what happens in my department is large(impact 1).
2. I have a great deal of control over what happens in my department(impact 2).
3. I have significant influence over what happens in my department(impact3).

Source: Spreitzer, G. M. 1995. Psychological empowerment in the workplace: Dimensions, measurements and validation. Academy of Management Journal, 3, 1442-1466.

Emotional Exhaustion (5-point scale: totally disagree to totally agree)

1. I feel emotionally drained from my work.
2. I feel used up at the end of the workday.
3. I feel fatigued when I get up in the morning and have to face another day on the job.
4. Working with people all day is really a strain for me.
5. I feel burned out from my work.
6. I feel frustrated by my job.
7. I feel I'm working too hard on my job.
8. Working with people directly puts too much stress on me.
9. I feel like I'm at the end of my rope.

Source: Maslach, C. and Jackson, S. E(1981).The measurement of experienced burnout. Journal of Organizational Behavior, 2(2), 99-113.
